# Supplementary material for: Improved Electrophoretic Separation to Assist the Monitoring of Bcl-xL Post-Translational Modifications
Source: Int J Mol Sci. 2019 Nov 8;20(22):5571. doi: 10.3390/ijms20225571 (PMC6888115; doi:10.3390/ijms20225571)
Supplement: Supplementary file 1 [file ijms-20-05571-s001.pdf]

Suppl Figure 1

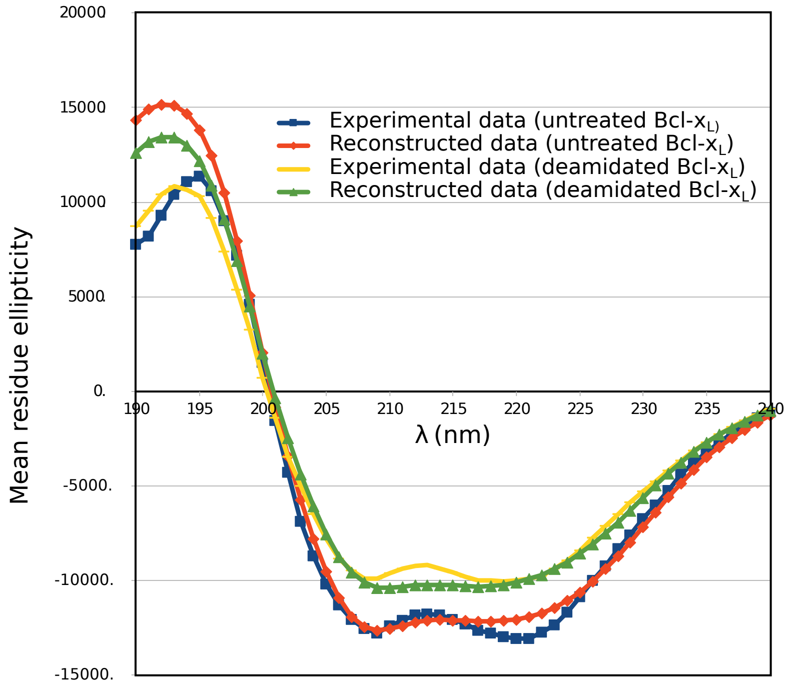

To calculate secondary structure fractions in untreated Bcl-xL and chemically deamidated Bcl-xL (pH=10 treatment), experimental CD spectra were analyzed with DichroWeb software using the SELCON3 program with SP175 reference dataset. Graph plots experimental and reconstructed data to show the accuracy of the calculation method chosen.
